# AI and digital assistive technologies and reproductive rights among women with disabilities in Egypt: a qualitative SWOT study

**DOI:** 10.3389/fsoc.2026.1925235

**Published:** 2026-08-31

**Authors:** AbdelMajeed Ahmed Hendy, Mastur Rehim, Raouf Kaouache, Ouassila Yaiche

**Affiliations:** 1 Department of Sociology, College of Arts, Humanities and Social Sciences, University of Al Dhaid, Sharjah, United Arab Emirates; 2 Department of Population Studies, Faculty of Arts, Minia University, Minya, Egypt

**Keywords:** artificial intelligence, assistive technologies, reproductive health, reproductive rights, SWOT analysis, women with disabilities

## Abstract

**Introduction:**

This study examined the potential contribution of artificial intelligence (AI)-supported and digitally enabled assistive technologies to reproductive-health-related access among women with disabilities in Minya Governorate, Egypt. It focused on access to reproductive healthcare services and information and on anticipated informed decision-making.

**Methods:**

An exploratory qualitative case-study design was used. In-depth interviews were conducted with eight never-married women aged 15–49 years with physical (*n* = 4) or visual (*n* = 4) disabilities. The study was guided by feminist theory, human-centered AI, and socio-technical systems theory, and the interview data were analyzed using the SWOT framework.

**Results:**

Participants perceived smart wheelchairs, prosthetic limbs, screen readers, smart canes, and AI-enhanced glasses as supporting mobility, navigation, information access, and potential access to healthcare services. Women with physical disabilities primarily emphasized mobility, whereas women with visual disabilities emphasized navigation and information access. Key barriers included high costs, limited maintenance services, privacy concerns, inaccessible infrastructure, insufficient healthcare-provider competencies, economic dependency, and social stigma.

**Discussion:**

AI-supported and digitally enabled assistive technologies may create enabling conditions for reproductive-health-related access and anticipated informed decision-making. However, the findings do not demonstrate that reproductive rights or improved health outcomes were directly achieved. Their perceived usefulness depended on supportive economic, institutional, infrastructural, familial, and sociocultural conditions. Given the small purposive sample and exploratory design, the findings are context-specific and should not be broadly generalized.

## 1 Introduction

Artificial intelligence (AI) technologies have increasingly transformed healthcare delivery and created opportunities to improve healthcare quality, communication, and patient access to services. The application of AI technology to support reproductive health may facilitate access to reproductive health information, improve communication between patients and healthcare providers, and support informed health-related decision-making. Despite AI’s widespread use and its benefits to society at large, there is an overall lack of research on how AI-supported and digitally enabled assistive technologies may support the reproductive rights of women with disabilities, especially in low-income countries or resource-poor areas.

Reproductive rights encompass access to reproductive healthcare services, reproductive health information and education, and the ability to exercise self-determination in reproductive health matters. They also include reproductive freedom concerning procreation, parenting, marriage, family formation, conception, and childbirth and are closely connected to equality, autonomy, dignity, privacy, and social inclusion. While reproductive rights encompass marriage, family formation, conception, and childbirth, this study focuses on access to healthcare resources and reproductive health knowledge to support informed decision-making. Within this study, improved mobility, information access, and independence are treated as potential enabling conditions for reproductive rights rather than as direct evidence that reproductive rights were achieved or improved.

The technical aspects of assistive technology devices for individuals with a disability can be significantly different. Tools that are conventional (i.e., mechanical) offer users either manual assistance or fundamental functionality, whereas digital assistive tools use one or more of the following technical elements; electronic components, software applications, sensors to detect physical movement or changes in an environment, sensory-based adaptations that react based on user input, and/or artificial intelligence (AI)-enabled tools using techniques such as machine learning, computer vision, natural language processing, or decision-making. Because participant-reported technology was not always AI-enabled (e.g., wheelchairs, screen readers), the researchers used a general term of “AI-supported and digitally enabled assistive technologies.” The researchers examined how these technologies were functionally classified with regard to their use in providing users with mobility, navigation, text-to-speech, environmental recognition, and accessing digital information.

This study addresses these gaps by examining the potential contribution of AI-supported and digitally enabled assistive technologies to creating enabling conditions for reproductive rights among women with physical and visual disabilities in Minya Governorate, Egypt. Specifically, the study explores how the accessibility functions of these technologies may contribute to: (1) improving access to reproductive healthcare services and resources; (2) facilitating access to reproductive health information and educational messages; and (3) supporting informed reproductive health decision-making. In addition, the study identifies the strengths, weaknesses, opportunities, and threats associated with these technologies from the perspective of women with disabilities. Because all participants were never married, these dimensions primarily concern anticipated needs, perceived accessibility, and expected future reproductive decision-making rather than direct experiences of pregnancy, childbirth, contraception, or family-planning service use.

The study makes three contributions. First, it provides context-specific qualitative evidence regarding the perceived relationship between AI-supported and digitally enabled assistive technologies and potential reproductive-health-related opportunities among women with disabilities in Upper Egypt. Second, it demonstrates the application of SWOT analysis as a framework for assessing assistive technologies in the context of reproductive health and disability. Third, it offers tentative, context-specific recommendations that may inform policymakers, healthcare providers, disability-support organizations, and technology developers seeking to support access to reproductive health resources and social inclusion for women with disabilities.

## 2 Research methodology: AI and digital assistive technologies and reproductive rights among women with disabilities

### 2.1 Research problem

Women with disabilities possess the same reproductive rights as other members of society, including the right to access reproductive healthcare services and resources, obtain reproductive health information and education, and make informed decisions regarding their reproductive health. Technological developments in AI-supported and digitally enabled assistive technologies have created potential opportunities to improve accessibility, communication, mobility, and knowledge gain for people with disabilities. Yet, it is still not clear to what extent these technologies can be leveraged to create enabling conditions for the exercise of reproductive rights among women with disabilities. In particular, improvements in general mobility, navigation, or information access do not necessarily demonstrate direct improvements in reproductive healthcare access or the substantive exercise of reproductive rights.

Three principal gaps frame this research problem. First, an empirical gap exists because limited evidence has examined how women with disabilities in Upper Egypt perceive the relationship between AI-supported and digitally enabled assistive technologies and access to reproductive-health-related services, information, and decision-making. Second, a methodological gap arises from the limited inclusion of women with disabilities in qualitative reproductive health research, particularly studies examining differences between physical and visual disabilities. Third, an analytical gap remains because feminist perspectives on reproductive rights have rarely been integrated with human-centered AI and socio-technical systems approaches to examine how technology interacts with poverty, family dependency, infrastructure, institutional practices, and social stigma.

This study uses an exploratory qualitative approach to examine the perceived opportunities, strengths, limitations, and risks associated with AI-supported and digitally enabled assistive technologies in relation to reproductive-health-related access among women with physical and visual disabilities in Minya, Egypt. The use of an exploratory method is suitable as there is very little previous literature that examines the interaction between AI and digital assistive technologies, disability, and reproductive rights. Because all participants were never-married women, the study primarily examines anticipated reproductive health needs, perceived barriers, information-seeking experiences, and expectations regarding future reproductive decision-making rather than direct experiences of pregnancy, childbirth, contraception, or family-planning service use. This study does not seek to test any causal links or establish that reproductive rights were directly achieved through technology use, but rather to develop context-specific knowledge, identify emergent themes, and create a base for future studies and policy development (Stebbins, 2001).

### 2.2 Research questions

This study is guided by the following research questions:

1. What perceived opportunities do AI-supported and digitally enabled assistive technologies create for women with physical and visual disabilities in accessing reproductive healthcare services, resources, information, and educational messages?
2. How do participants perceive the relationship between the reported functions of these technologies—such as mobility, navigation, and information access—and their potential access to reproductive health resources and informed reproductive health decision-making?
3. What internal weaknesses or functional limitations of AI-supported and digitally enabled assistive technologies constrain their potential role in supporting access to reproductive health services, information, and decision-making among women with disabilities?
4. What external environmental, institutional, economic, familial, and socio-cultural threats restrict the effective use of AI-supported and digitally enabled assistive technologies as potential enabling resources for reproductive rights among women with disabilities?

### 2.3 Literature review

Research on artificial intelligence, disability, and reproductive rights can be organized into four major themes. The first theme concerns the potential contribution of AI to reproductive health access, communication, and decision-making. According to the World Health Organization (2024), artificial intelligence may enhance the autonomy of individuals who are seeking to make decisions about reproductive health, increase access to reproductive-health-related services, and offer information to help individuals make informed decisions regarding their health. Additionally, Gbagbo et al. (2024) argue that AI may support reproductive health and rights through reducing inequalities and improving service delivery for women, although important ethical issues must be addressed when using this technology. Furthermore, Kakkar et al. (2025) indicate that integrating AI into healthcare technologies may improve communication between patients and their healthcare providers and increase patient access to health-related services. However, this literature primarily concerns healthcare systems and broader patient populations and provides limited evidence regarding women with disabilities or the direct exercise of reproductive rights.

The second theme is concerned with the intersection of disability and reproductive rights. Vaidya (2015) emphasizes that it is important to recognize the reproductive rights of women with disabilities as a means to promote empowerment, bodily autonomy, and individual decision-making. Scholars in the field of disability studies also suggest that many barriers experienced by people with disabilities arise not only from impairment but also from social, environmental, or institutional exclusion that limits the ability to participate and have access to resources (Shakespeare, 2014). Courduriès and Herbrand (2014) identify assisted reproductive technologies as an area in which gender, family, and reproductive decision-making intersect with the broader social context. Chiweshe et al. (2017) argue that reproductive justice requires women to be able to make reproductive health decisions within their particular social and cultural circumstances. Nevertheless, many of these same women are limited in accessing those same reproductive rights due to social, economic, governmental, and service-related restrictions (Grzanka and Frantell, 2017; Abarbanell, 2020). Such issues have also been observed in the Arab region. In addition to legal rights, Reproductive Rights are contingent upon a woman’s ability to have access to resources, supportive institutions and socially inclusive environments according to Rehim (2024). In addition to social exclusion, women with disabilities in Libya experience other barriers to exercising rights and participating in society including: service deprivation, social isolation, dependence on others for financial support and lack of institutional support.

AI and disability inclusion is the third theme. Evidence suggests that emerging digitally enabled assistive tools may improve communication accessibility, quality of life, mobility, navigation for people with disabilities through use of AI driven systems (Khan, 2024; Alsaleh, 2026). Participation can also be supported by reducing barriers of information access, communication, mobility, navigation. However, the literature also identifies several risks, including algorithmic bias, exclusion, discrimination, high costs, and limited access to technology (Hasan et al., 2025; El Morr et al., 2024; Zarra et al., 2023; Lourenço et al., 2025). The concerns presented here relate to a more general critique of AI systems, such that they could perpetuate many current social inequalities and modes of exclusion if the technological design does not consider the needs and perspectives of those who have been marginalized (Benjamin, 2019). It is also important to recognize how different types of assistive technologies can be characterized technologically, and therefore, do not represent the same type of product or service. Therefore, it is imperative to make this distinction when determining whether observed benefits were due to the presence of artificial intelligence, or by virtue of being an assistive technology.

Reproductive rights are also shaped by a number of other factors including the social and institutional environment. For example, effective access to reproductive health care is dependent upon healthcare providers that have received appropriate training and work in an institution that supports their efforts. In addition, it is dependent upon individuals being able to receive services in private, having available infrastructure such as transportation and clinics, financial resources for care and procedures, positive support from families, and the lack of discrimination against marriage and motherhood based on disability (Romero et al., 2016; Grzanka and Frantell, 2017).

Evidence for these challenges comes from the Middle East and other resource-challenged regions. An educational intervention involving deaf and hearing-impaired females in Saudi Arabia was shown to improve cervical cancer related knowledge and beliefs regarding reproductive health; this shows the need for both appropriate reproductive health education and accessible methods of communicating (El Sayed et al., 2022). Women with disabilities in Ethiopia reported a number of barriers to receiving sexual and reproductive healthcare including lack of funds, difficulty traveling, barriers due to organization, lack of awareness of existing services, and negative attitudes toward them in their communities; on the other hand they reported greater access when there was support from others, they had more education, they were treated positively by healthcare workers, and they had confidence in themselves (Alemu et al., 2024). A similar qualitative study conducted in Ghana also showed that limitations in accessing maternal care included: lack of accessibility in travel to healthcare facilities and lack of adequate training or knowledge on disability issues for healthcare providers, negative attitudes of providers, and assumptions made by providers regarding the roles of women with disabilities in reproduction (Ganle et al., 2016).

Although legal recognition of disability rights exists through laws such as the disability act (Law No. 10 of 2018), which was enacted by Egypt’s government to protect and promote the rights of people with disabilities, challenges remain regarding accessibility for reproductive healthcare services within health care facilities, particularly in resource-constrained regions such as Upper Egypt. Therefore, while formal legal recognition of the rights of people with disabilities is provided for under the law, it does not necessarily translate into accessible, confidential, or disability-sensitive reproductive healthcare in practice.

Overall, AI-supported and digitally enabled assistive technologies may improve mobility, navigation, communication, and information access, but these benefits should not be interpreted as direct evidence of reproductive healthcare use or reproductive autonomy. Limited research has examined these relationships among women with disabilities in Egypt or applied SWOT analysis to identify the technological and contextual factors shaping reproductive-health-related access. This study addresses this gap among women with physical and visual disabilities in Minya Governorate.

### 2.4 Theoretical framework

This research draws upon three theoretical frameworks: feminist theory, human-centered AI, and socio-technical systems theory. These theories form a base for examining whether AI-supported and digitally enabled assistive technologies may create enabling conditions for women’s reproductive rights. Feminist theory informs the analysis of bodily integrity, autonomy, equality, diversity, and access to resources; human-centered AI informs the examination of accessibility, flexibility, and responsiveness; and socio-technical systems theory guides the interpretation of interactions among technology, individuals, families, institutions, infrastructure, and the social environment.

Modern liberal feminism advocates greater reproductive freedom through equality, individual self-determination, bodily integrity, and personal privacy (Brown, 2005, pp. 88–96). Feminist literature describes reproductive rights in terms of power and access to resources (Correa and Petchesky, 1994). The contemporary feminist literature has further defined reproductive justice as a paradigm for understanding how reproductive rights are tied to broader social inequalities in access and structure (Ikemoto, 2018). The term power is used here to define an individual’s capacity to obtain information, express preferences, and make informed choices about their own reproductive health. Resources refer to the physical, informational, technological, and institutional conditions required by individuals to carry out these reproductive choices safely.

Correa and Petchesky (1994) articulate reproductive rights in four interrelated principles. The first principle is bodily integrity, which removes barriers for individuals to reach their maximum physical and reproductive potential. The second principle is personhood or self-governance and independent decisions as it pertains to reproductive choices. The third principle is equality and removes inequities in availability of health care services and resources. This includes inequities felt by women with disabilities. The fourth principle is diversity and acknowledges the differences among women based on culture, religion, beliefs, family situations, and health concerns.

These four principles informed the interview domains, coding, and interpretation used in this analysis. Bodily integrity guided the examination of perceived mobility, physical-access, and infrastructural barriers to reproductive healthcare. Personhood guided the examination of participants’ access to reproductive health information, expression of preferences, and anticipated decision-making. Equality guided the examination of perceived disparities in healthcare accessibility, privacy, provider practices, and access to assistive technologies. Diversity was taken into account when comparing women with physical and visual disabilities and interpreting social, cultural, familial, economic, and environmental influences. The approach taken here is similar to what scholars have argued when they speak about reproductive justice, where they argue that having rights without resources is meaningless. Chrisler (2013, p. 10) argues that reproductive justice emerges through the interaction of rights, resources, and access. In the context of the present study, AI-supported and digitally enabled assistive technologies may be understood as potential enabling resources that may facilitate access to reproductive healthcare services, reproductive health information, and anticipated informed decision-making among women with disabilities.

Within this framework, AI-supported and digitally enabled assistive technologies are conceptualized as potential resources that may improve mobility, navigation, communication, and information access, thereby creating opportunities to support access to reproductive healthcare and anticipated reproductive decision-making. This relationship is treated as indirect and does not establish that reproductive rights were directly achieved through technology use. Consequently, the study also adopts a human-centered AI perspective, which emphasizes that AI technologies should be designed to enhance human capabilities, improve wellbeing, and respond to users’ needs while addressing ethical and social concerns (Xu, 2019).

In the context of this study, the human-centered AI approach is reflected through three dimensions. Accessibility refers to the extent to which women with disabilities can independently use AI-supported and digitally enabled assistive technologies and whether these technologies reduce barriers to mobility, navigation, communication, or information access. Flexibility refers to the ability of these technologies to function across different physical and social environments, including homes, health centers, roads, and public spaces. Responsiveness refers to the degree to which these tools address disability needs related to access to reproductive healthcare and information about reproductive health. These three dimensions informed the coding and interpretation of participants’ accounts of technological strengths and weaknesses.

In addition, the research uses sociotechnical system theories (STS), where it is stressed that any technological achievements result from the interaction between technological, social, and ecological systems, as opposed to the achievement of results by technological means alone (Abbas and Michael, 2025; Clegg et al., 2017). According to the theory, the perceived usefulness of AI-supported and digitally enabled assistive technologies depends not only on the devices themselves but also on affordability, family support, maintenance services, provider competencies, privacy protections, healthcare accessibility, transportation, infrastructure, and socio-cultural attitudes.

This approach is especially significant within the scope of the adopted SWOT analysis, which supports the examination of internal technology-related strengths and weaknesses and external economic, familial, institutional, infrastructural, social, and cultural opportunities and threats. Therefore, the theory of socio-technical systems can be regarded as a suitable approach to explaining how different forces interact to create or constrain enabling conditions for reproductive rights among women with disabilities.

This study’s theoretical framework has its own constraints. First, liberal feminist perspectives may not fully capture reproductive choices shaped by family connections, cultural norms, and local expectations in social settings. Second, human-centered AI approaches frequently assume that users have sufficient autonomy, technological access, and control over their circumstances to use new technologies. That is not true in many situations with limited resources. Third, while the framework identifies how disability, technology, and social conditions interact, it does not account for every dimension of inequality that could affect reproductive rights. The analysis therefore considers the intersecting influence of gender, disability type, unemployment, economic dependency, geography, family relationships, institutional exclusion, and social stigma without claiming to measure each dimension separately.

Another issue concerns the tension between feminist concepts of individual autonomy and the use of family support during participant interviews. Although autonomy is highly valued in feminist theory, mothers or sisters were present during the interviews to support communication, accessibility, and participant comfort. This practice was employed to increase participant comfort and accessibility. However, family involvement may have both supported participation and influenced the expression of views concerning sexuality, marriage, contraception, abuse, and reproductive health decision-making. It is therefore interpreted as a potential source of support as well as a methodological influence on participant responses.

### 2.5 Research methodology

This study applied an exploratory qualitative case-study design through in-depth interviews. The case-study method was considered suitable because the study sought to provide an in-depth description of how AI-supported and digitally enabled assistive technologies may create enabling conditions for reproductive-health-related access and anticipated decision-making among women with disabilities within a specific social and geographical environment. The study did not seek to establish causal effects or demonstrate that reproductive rights were directly achieved through technology use.

The study was carried out in Minya City, Minya Governorate, Egypt, and focused on women with physical and visual disabilities who used AI-supported, digitally enabled, sensor-supported, or technologically enhanced assistive devices. A purposive sampling method was employed to identify information-rich cases capable of providing detailed insight into participants’ perceptions of the relationship between assistive technology use and reproductive-health-related access. The documented participant-selection characteristics included being a woman aged 15–49 years, having a physical or visual disability, and using at least one of the assistive technologies examined in the study. Participants were identified with the guidance and support of professionals specializing in prosthetics, orthotics, optometry, and assistive technologies.

The sample comprised eight never-married women aged 15–49 years, with a mean age of 33 years. Four had physical disabilities and four had visual disabilities. The purposively selected cases provided information-rich, context-specific accounts suitable for an exploratory qualitative study and were not intended to be statistically representative.

Recruitment continued until the research team judged that the interviews provided sufficient information richness for the narrowly defined exploratory aims and that additional interviews were not producing substantially new SWOT themes (Table 1).

**Table 1:** Characteristics of study participants.

Participant	Disability type	Assistive technology used	Education level	Employment status
P1	Physical	Smart wheelchair	University	Unemployed
P2	Physical	Smart wheelchair	Intermediate	Unemployed
P3	Physical	Prosthetic limb	University	Unemployed
P4	Physical	Prosthetic limb	University	Unemployed
P5	Visual	Screen reader + smart cane	University	Unemployed
P6	Visual	Screen reader + smart cane	University	Unemployed
P7	Visual	Screen reader + smart cane	Master’s degree	Unemployed
P8	Visual	AI-enhanced glasses + VoiceOver	University	Unemployed

All participants used a heterogeneous range of directly AI-enhanced, digitally enabled, sensor-supported, signal-responsive, or technologically enhanced assistive technologies to overcome disability-related barriers in their daily lives. For analytical purposes, Eyedaptic glasses were classified as participant-reported AI-enhanced visual-assistance technology; screen readers and VoiceOver as digitally enabled text-to-speech technologies; smart canes as sensor-supported navigation devices; prosthetic limbs as technologically enhanced, signal-responsive mobility devices; and smart wheelchairs as technologically enhanced mobility devices. The exact device models, software versions, proprietary algorithms, and degree of AI integration were not independently verified. Accordingly, the study does not assume that every reported technology inherently incorporated artificial intelligence. Data collection was conducted by female researchers enrolled in master’s and doctoral programs under the supervision of the principal researcher.

The interview questions were designed according to the research objectives, the concept of reproductive rights, and the SWOT analysis approach. The interview guide covered assistive technology use, mobility and navigation, access to reproductive healthcare services, reproductive health information, anticipated decision-making, privacy, affordability, infrastructure, family support, healthcare-provider practices, and social stigma.

The complete semi-structured interview guide used during data collection is provided as Supplementary material 1. The interviews were conducted in Arabic, and the supplementary guide is presented in English translation. The guide was reviewed by the research team for clarity, relevance, sensitivity, and alignment with the study objectives before data collection; however, it was not formally pilot-tested.

Data were collected in 2025 in Minya City, Egypt. The interviews were conducted in Arabic, and each interview lasted approximately 30–45 min. With participants’ verbal permission, the interviews were audio-recorded and transcribed verbatim in Arabic. Quotations selected for inclusion in the English-language manuscript were translated into English by a researcher with translation experience and independently checked by a bilingual reviewer to ensure accuracy and preserve the participants’ intended meanings. Before data collection, the female researchers received training in qualitative interviewing, disability-sensitive communication, research ethics, confidentiality, informed consent, and the discussion of sensitive reproductive-health topics. At the time of data collection, participants had used their reported assistive technologies for approximately 2 months.

Family members were present during all eight interviews at the researchers’ request to support communication, accessibility, and participant comfort. Interviews could be conducted privately if desired; however, family members provided no answers to the researchers’ questions nor contributed anything substantively to their respective interviews. As such, the communication-supportive strategy used here has some similarities to the Sisterhood Method (Graham et al., 1989), which provides a mechanism for obtaining information when there are difficulties communicating directly. However, it was not utilized as a methodology within this research. Nonetheless, the presence of family members attending these interviews may have impacted how participants expressed their views on sexual matters, marriage, birth control, sexual/physical abuse, privacy, and reproductive health decisions and as such is recognized as an important methodological limitation.

The collected data were analyzed using the SWOT framework (Strengths, Weaknesses, Opportunities, and Threats) as an organizing framework for qualitative interpretation. SWOT was selected because it allows the systematic examination of both internal characteristics of AI-supported and digitally enabled assistive technologies and external economic, familial, institutional, infrastructural, social, and cultural factors influencing their perceived usefulness.

The analytical process involved four stages. First, the reproductive-rights dimensions examined in the study were identified, including access to healthcare services and resources, access to reproductive health information, and informed reproductive health decision-making. Second, interview data were reviewed, compared across participants and disability groups, and organized according to recurrent and emerging themes related to strengths, weaknesses, opportunities, and threats. The reproductive-rights dimensions and SWOT categories provided the initial analytical structure, while issues repeatedly identified in participants’ accounts were retained as qualitative themes. Third, findings were synthesized into a SWOT matrix to identify key enabling and constraining factors associated with potential access to reproductive healthcare services, reproductive health information, and anticipated decision-making. Finally, the SWOT matrix was used to develop strategic recommendations and an intervention framework designed to strengthen opportunities, address weaknesses, and mitigate threats. Participant quotations were used, where available, to illustrate key themes and maintain a visible connection between participants’ accounts and the resulting interpretations.

The qualitative analysis was conducted manually by all four researchers without the use of qualitative analysis software. Each transcript was reviewed and coded according to the reproductive-rights dimensions and the SWOT categories, while recurrent issues emerging from participants’ accounts were retained as qualitative themes. Coding differences were reviewed by the principal researcher and resolved through discussion and consensus among the research team. Peer debriefing and collaborative review were conducted among all four researchers to evaluate the consistency of the codes, themes, and interpretations. Member checking was conducted by presenting the preliminary interpretations to participants and inviting them to confirm, clarify, or correct the researchers’ understanding of their responses. This process was facilitated by the research team. Quotations were selected to illustrate the principal themes, represent both disability groups, and maintain a clear connection between participants’ accounts and the researchers’ interpretations.

The analysis compared the mobility-related experiences of women with physical disabilities with the navigation- and information-access experiences of women with visual disabilities. It also distinguished participants’ reported perceptions from the researchers’ interpretations and did not treat the findings as statistically representative or causally established. The principal researcher is male, while data collection was conducted by female researchers enrolled in master’s and doctoral programs. This arrangement was intended to reduce gender-related discomfort when discussing reproductive health topics. However, the researchers acknowledge that interviewer characteristics, family presence, and the broader sociocultural context may have influenced participants’ responses and the interpretation of the findings.

### 2.6 Ethical considerations

The study received ethical approval from the relevant institutional ethics committee and was conducted in accordance with the ethical principles and standards governing scientific research.

Verbal informed consent was obtained from all adult participants before the interviews. One participant was younger than 18 years; for this participant, verbal permission was obtained from a parent or legal guardian, and the participant provided verbal assent. Disability status was not treated as evidence of impaired decision-making capacity, and adult participants provided consent on their own behalf.

Participation in the study was voluntary. Information on the intent behind the research as well as the use of data for research and publication purposes were provided to participants. Confidentiality was protected through de-identification of all personal identifiers within the interview records and use of participant codes to report findings. Due to the sensitive nature of reproductive health issues and inclusion of women with disabilities, privacy, respect and participant comfort were considered when conducting the interviews. Mothers or sisters were present during the interviews to support communication, accessibility, and participant comfort. Participants were advised that they had the option to refuse to answer any questions presented and/or withdraw from participating at any point during the interview.

## 3 Analysis of participants’ responses using the SWOT framework

### 3.1 Perceived strengths of assistive technologies and opportunities for supporting reproductive rights

Participants’ accounts suggested that assistive technologies may create potential enabling conditions for reproductive rights through three interlinked pathways: improved mobility and potential access to healthcare facilities, improved access to reproductive health information, and support for anticipated reproductive health decision-making. These pathways represent perceived opportunities rather than direct evidence that reproductive rights, reproductive healthcare access, or reproductive health outcomes were achieved or improved. The perceived benefits differed according to disability type: women with physical disabilities primarily emphasized mobility-related functions, whereas women with visual disabilities emphasized navigation and information-access functions.

Participants with physical disabilities (P1–P4) reported using technologically enhanced mobility devices: two used smart wheelchairs and two used signal-responsive lower-limb prosthetic devices. They described these technologies as supporting greater mobility independence and reducing reliance on family members or caregivers for movement. Improved mobility was perceived as creating greater opportunities to reach healthcare facilities and seek reproductive health information when needed.

One participant using a technologically enhanced wheelchair reported that limited mobility had previously made it difficult for her to leave home or reach healthcare and educational facilities. She stated: “Before the smart wheelchair, I always relied on my family any time I wanted to go out. I am now able to move freely and visit places I could not easily get to before, such as health facilities.” (Participant using a smart wheelchair). Another participant using a signal-responsive lower-limb prosthesis reported that the device increased her confidence and supported more independent movement within her immediate environment.

For participants with visual disabilities (P5–P8), AI-enhanced, digitally enabled, and sensor-supported assistive technologies were perceived as primarily supporting information access and independent navigation. Three participants with complete vision loss used screen readers, Braille-related tools, and sensor-supported smart canes, while one participant with severe visual impairment used participant-reported AI-enhanced Eyedaptic glasses and VoiceOver. These technologies were described as facilitating access to information that might otherwise require assistance from family members or service providers. Participants perceived these technologies as facilitating access to educational content related to reproductive health, family planning, menstrual health, disease prevention, and healthcare services.

The findings indicate that visually impaired participants relied more heavily on information-access technologies than participants with physical disabilities. While women with physical disabilities primarily emphasized mobility-related benefits, women with visual disabilities highlighted the importance of information accessibility and independent learning. Technologies such as screen readers and participant-reported AI-enhanced Eyedaptic glasses were described as enabling participants to access written materials, health information, and digital resources with greater independence. One participant using screen-reader technology explained: “I can listen to information through the screen reader whenever I want. I do not always need someone else to read things for me, and I can repeat the information as many times as necessary.” (Participant using screen-reader technology). However, the ability to access digital content did not establish that the reproductive health information obtained was accurate, clinically appropriate, accessible in language and format, or tailored to participants’ individual needs.

One participant with a physical disability described combining family-based reproductive health knowledge with information obtained from healthcare facilities and other sources. As one participant stated: “I receive information from my mother, but I also prefer to verify information by visiting healthcare centers and searching for additional information whenever possible.” (Participant with a physical disability).

This statement suggests that improved accessibility may allow women with disabilities to diversify their information sources rather than relying exclusively on family-based knowledge. Access to multiple sources may strengthen perceived confidence in anticipated reproductive health decision-making; however, the account does not demonstrate actual independent decision-making or the substantive exercise of reproductive autonomy.

All four participants with physical disabilities (P1–P4) identified needs for information concerning physical and reproductive changes, available reproductive health services, safe motherhood, marriage and spouse selection, premarital screening, and sexually transmitted infections. All four participants with visual disabilities (P5–P8) identified needs concerning menstrual hygiene, the onset and management of menstruation, protection from sexual assault and harassment, the reproductive and sexual health risks of female genital mutilation, and early detection of reproductive-system diseases and breast tumors. These accounts show that participants understood reproductive rights as encompassing not only potential access to healthcare facilities but also access to relevant information, education, privacy, and support for informed future decision-making.

The findings also suggest that participants perceived AI-supported and digitally enabled assistive technologies as supporting self-confidence, autonomy, and social participation. Participants often linked technological support with greater mobility, improved self-reliance, and potentially greater engagement with healthcare systems and community resources. These participant-reported experiences are consistent with human-centered AI principles, which focus on enhancing human capabilities and wellbeing through accessible and adaptive technologies (Xu, 2019). Nevertheless, they indicate perceived capability enhancement rather than direct evidence of substantive reproductive autonomy. Because all participants were never married, their accounts primarily reflected anticipated rather than lived reproductive-health experiences.

### 3.2 Perceived weaknesses of assistive technologies and threats to supporting reproductive rights

Participants’ concerns largely centered on affordability, maintenance, accessibility, and the availability of technical support. All eight participants (P1–P8) reported that the acquisition, repair, and maintenance costs of the assistive technologies were beyond what they could independently afford. One participant explained: “The technology really does help me a great deal, however the price is very excessive. When the equipment malfunctions, I have to spend a large amount of money to repair it, plus at times replacement parts cannot be found.” (Participant using assistive technology).

The findings also illustrate the intersection of gender, disability, unemployment, economic dependency, and access to technology. All participants were unemployed and economically dependent on their family members; therefore, access to assistive technologies depended on household financial resources and continued family support. As a result, the advantages of assistive technology were determined not solely by the technologies themselves but also by participants’ socioeconomic circumstances and family dependency. Technology therefore appeared to reduce some forms of mobility or information dependency while potentially creating or reinforcing other forms of dependency on family financing, maintenance services, spare parts, and technical support. Furthermore, the intersection of disability, gender, unemployment, and economic dependency suggests that technological provision alone is unlikely to remove barriers to reproductive rights without complementary measures addressing social equity, economic opportunities, and institutional support.

Privacy emerged in two related forms: digital data privacy when using assistive devices and reproductive-health confidentiality within healthcare settings. Participants with visual disabilities raised concerns about who could access personal information stored or transmitted through communication devices, information-search tools, screen readers, and other digital assistance technologies. Some participants specifically noted the need for increased protections via laws and institutions to safeguard their personal data. One visually impaired participant stated: “I am worried about who has access to the information being held on my devices. More than half of the time, I do not know how to make sure my information will be private. “(Participant with a visual disability).

Participants also described concerns about privacy during healthcare consultations, including the presence of unnecessary individuals in service rooms, limited transparency concerning examination procedures, and uncertainty about how providers managed personal health information. These concerns could discourage full disclosure of sensitive reproductive health information.

The study found additional concerns about the preparedness of healthcare professionals to work with women with disabilities in a manner that was effective. All eight participants (P1–P8) expressed concern that some healthcare providers lacked the disability-related knowledge, communication skills, and professional competencies needed to provide appropriate reproductive health education, counseling, privacy, and support. These findings are consistent with the results of Romero et al. (2016), who emphasize the need for professional education/training and disability sensitive reproductive health care services.

Infrastructure-related barriers were also identified by some participants. Participants described challenges such as unpaved roads, poorly designed or inaccessible sidewalks, limited accessible transportation, and a lack of disability-friendly public facilities. While AI-supported and digitally enabled assistive technologies enhanced mobility and independence, participants noted that these benefits were often limited by environmental conditions that remained outside their control.

All eight participants (P1–P8) identified social stigma as an important external barrier, particularly the belief that women with disabilities are unlikely or unable to marry, become mothers, or exercise reproductive roles. As one participant stated: “some people believe that women with a disability cannot get married and/or have children. That sometimes causes some people to treat us poorly when we go looking for information and/or services.” (Participant with a disability). These accounts suggest that negative social attitudes may affect the reproductive rights and social inclusion of women living with disabilities, supporting the feminist theory about reproductive rights (Correa and Petchesky, 1994). This finding is supported by the research conducted in Libya, where women living with disabilities experienced social exclusion, lack of services and institutional support, preventing them from exercising their rights (Rehim, 2024). In addition, the findings suggest that technological accessibility alone may be insufficient to support the exercise of reproductive rights without social acceptance, institutional inclusion, and respectful healthcare practices.

Overall, the potential contribution of assistive technologies depended on affordability, family support, maintenance services, provider competencies, privacy, infrastructure, and social stigma. These technologies did not automatically result in reproductive healthcare use or substantive reproductive justice.

## 4 Discussion

This exploratory study suggests that AI-supported and digitally enabled assistive technologies may indirectly support reproductive-health-related access among women with disabilities. By improving mobility, navigation, and information access, these technologies may create opportunities to reach healthcare facilities, obtain reproductive health information, and prepare for future decision-making. However, these perceived opportunities do not demonstrate actual reproductive healthcare use or the direct exercise of reproductive rights.

The perceived benefits differed by disability type. Women with physical disabilities mainly emphasized improved mobility and reduced reliance on others, whereas women with visual disabilities emphasized navigation, independent learning, and access to digital information. This indicates that the usefulness of assistive technologies depends on the interaction between disability type, technological function, and environmental barriers.

Technology may also reduce some forms of dependency while reinforcing others. Although participants described greater mobility or information access, all remained unemployed and financially dependent on their families. Continued technology use therefore depended on affordability, maintenance services, spare parts, and family support. This supports socio-technical systems theory, which emphasizes that technological outcomes are shaped by interactions among users, institutions, infrastructure, and social conditions (Clegg et al., 2017; Abbas and Michael, 2025).

Participants also identified digital privacy concerns, limited confidentiality in healthcare settings, insufficient provider competencies, inaccessible infrastructure, and social stigma. These findings support the view that reproductive justice depends not only on technology or formal rights but also on accessible services, privacy, institutional support, economic resources, and social inclusion (Correa and Petchesky, 1994; Grzanka and Frantell, 2017; Rehim, 2024).

The findings are consistent with previous empirical research from comparable resource-constrained settings. The transportation, infrastructure, organizational, and healthcare-provider barriers reported by participants correspond with the barriers identified by Alemu et al. (2024) in Ethiopia and Ganle et al. (2016) in Ghana. The importance participants placed on accessible reproductive-health information also supports El Sayed et al. (2022), who demonstrated the value of disability-adapted reproductive-health education among deaf and hard-of-hearing women in Saudi Arabia. The present study extends this literature by examining a broader range of mobility, navigation, and information-access technologies and by highlighting the specific interaction of unemployment, family financial dependency, infrastructural barriers, and anticipated reproductive-health needs among women with disabilities in Upper Egypt.

Because all participants were never married, many accounts reflected anticipated reproductive health needs rather than direct experiences of pregnancy, contraception, childbirth, or family-planning services. The findings should therefore be interpreted as context-specific perceptions and expectations. Overall, assistive technologies may serve as enabling resources, but their contribution to reproductive rights remains conditional on supportive economic, institutional, infrastructural, familial, and sociocultural conditions.

## 5 Development of the SWOT matrix (strengths, weaknesses, opportunities, and threats)

Internal factors are those related to the technologies themselves, including their reported functionality, accessibility, flexibility, affordability, maintenance requirements, and data-related features, while external factors concern the environmental, institutional, economic, familial, infrastructural, social, and cultural contexts in which the technologies are used. Strengths and weaknesses were classified as internal factors, whereas opportunities and threats were classified as external factors. The reported capacity of assistive technologies to support mobility, navigation, and information access was classified as a strength because these functions relate to characteristics of the technologies themselves. Barriers related to infrastructure, healthcare-provider preparedness, privacy within healthcare settings, accessible transportation, and cultural attitudes were classified as threats because they arise primarily from the surrounding institutional, social, and environmental context rather than from the technologies themselves (Table 2).

**Table 2:** SWOT matrix of AI-supported and digitally enabled assistive technologies as potential resources for reproductive-health-related access.

Strengths (internal positive factors)	Weaknesses (internal negative factors)
•Support independent mobility and reduce reliance on assistance from others (P1–P4).	•High acquisition costs (P1–P8).
•Support feelings of freedom, equality, and empowerment among women with disabilities.	•Scarcity of high-quality technologies with appropriate standardized specifications.
•Support self-confidence and independence.	•Limited availability of spare parts and technicians qualified to maintain the technologies (P1–P8).
•Provide flexibility in accessing sources of reproductive health information (P5–P8).	•Limited funding and financial support for users with low incomes.
•Provide flexibility in interacting with reproductive health information and educational resources (P5–P8).	•Limited guarantees for the protection and confidentiality of users’ information and data (P5–P8).

Opportunities (external positive factors).	Threats (external negative factors)
•Potentially facilitate access to healthcare facilities (P1–P4).	•Limited privacy while receiving consultations and information at healthcare facilities.
•Provide opportunities to receive advice and interact directly with healthcare providers.	•Limited knowledge, skills, and capabilities among some healthcare providers in addressing the needs of women with disabilities (P1–P8).
•Support self-education and information seeking through digital technologies (P5–P8).	•Infrastructure barriers, including unpaved roads, uneven sidewalks, inaccessible pathways, and unsuitable transportation.
•Facilitate access to information through audio descriptions of texts and printed materials (P5–P8).	•Cultural perceptions that hinder social inclusion and contribute to stigma and discrimination against women with disabilities (P1–P8).
•Potentially support informed reproductive health decision-making.	–

The matrix summarizes participants’ reported experiences and perceptions. The identified opportunities represent potential enabling conditions and should not be interpreted as evidence that reproductive healthcare access, independent decision-making, or reproductive rights were directly achieved. Not every assistive technology reported by participants necessarily incorporated artificial intelligence.

The SWOT analysis suggests that AI-supported and digitally enabled assistive technologies may create potential opportunities to support reproductive-health-related access through improved mobility, information availability, navigation, and anticipated decision-making. However, these findings also indicate that technology alone cannot ensure actual reproductive healthcare access, independent reproductive decision-making, or substantive reproductive justice. Factors such as cost, family economic dependency, limited maintenance services, inadequate infrastructure, privacy concerns, insufficient healthcare-provider competencies, and social stigma remain potential barriers to the exercise of reproductive rights among women with disabilities.

From a feminist perspective, the strengths identified in the matrix may be understood as potential enabling resources that support mobility, information access, self-confidence, and opportunities for future reproductive decision-making (Correa and Petchesky, 1994). However, the identified weaknesses and threats illustrate how formally recognized rights may remain difficult to exercise when women lack affordable technologies, accessible services, privacy, institutional support, and social acceptance. Similarly, consistent with Clegg et al. (2017) and Abbas and Michael (2025), the perceived usefulness of AI-supported and digitally enabled assistive technologies depends not only on their technical characteristics but also on interactions among users, families, healthcare institutions, infrastructure, economic conditions, and the broader social environment.

The SWOT matrix provides a useful structure for organizing participants’ accounts; however, it necessarily simplifies the complex relationships among technological, familial, social, economic, infrastructural, and institutional factors. The high costs of assistive technologies interacted with participants’ unemployment and financial dependence on their families, limiting their ability to acquire, maintain, or replace devices independently. Cultural stigma can also be strengthened by a lack of provider competences and a lack of accessible health care facilities. Therefore, future research should examine how these factors interact over time and whether their cumulative influence affects actual reproductive healthcare use, privacy, decision-making, and reproductive-rights outcomes. Because the present study is exploratory and based on eight never-married participants, the SWOT matrix should be interpreted as a context-specific analytical summary rather than as a comprehensive or generalizable model.

## 6 Proposed intervention strategy

From these findings, the intervention strategy will seek to enhance strengths while at the same time overcoming the identified weaknesses and threats. Because the study was exploratory and involved a small, context-specific sample, the proposed strategies should be regarded as tentative recommendations requiring further consultation, implementation, and evaluation.

### 6.1 Leveraging strengths to expand opportunities (SO strategy)

Participants’ accounts suggest that AI-supported and digitally enabled assistive technologies may support independent mobility, information access, self-confidence, and potential interaction with healthcare services. These strengths can be utilized to expand opportunities for reproductive health promotion among women with disabilities.

Particular attention should be given to integrating AI-supported assistive technologies into reproductive health education and service-delivery programs. Healthcare institutions and disability-support organizations can utilize these technologies to improve access to reproductive health information, facilitate communication with service providers, and strengthen informed decision-making. In addition, AI-enabled accessibility tools may be incorporated into digital health platforms and educational materials to ensure that reproductive health information is available in formats accessible to women with different types of disabilities.

### 6.2 Using opportunities to address weaknesses (WO strategy)

Government agencies, disability-support programs, and charitable organizations may consider developing subsidy mechanisms that improve access to assistive technologies for low-income women with disabilities. In addition, partnerships between technology manufacturers, healthcare institutions, and disability organizations could facilitate maintenance services, technical support, and the availability of spare parts. Such measures would help ensure the long-term sustainability of AI-supported assistive technologies and reduce the risk of service interruptions resulting from device malfunction.

### 6.3 Leveraging strengths to address threats (ST strategy)

Although AI technologies improve accessibility and independence, participants identified several external threats, including inadequate infrastructure, limited privacy within healthcare settings, and insufficient disability-related competencies among healthcare providers. In response to these issues, organizations providing reproductive healthcare should improve access by ensuring that facilities, consultation rooms, and digital systems are accessible to people with disabilities. The providers of reproductive healthcare services should also be trained on reproductive healthcare for people with disabilities and other issues including confidentiality, communication, and patient-focused healthcare.

### 6.4 Minimizing weaknesses and threats (WT strategy)

Stigma, discrimination, and perceptions related to the reproductive role of women with disabilities were recognized as major impediments to promoting reproductive rights.

Accordingly, awareness programs targeting families, healthcare providers, community organizations, and the wider public should be developed to promote disability inclusion and challenge discriminatory attitudes. Educational institutions, religious organizations, media platforms, and civil-society organizations may play an important role in fostering more inclusive understandings of disability, marriage, family life, and reproductive rights.

In addition, women with disabilities should be actively involved in evaluating reproductive health services and AI-supported assistive technologies. Their experiences can provide valuable feedback for healthcare providers, policymakers, and technology developers seeking to improve accessibility, privacy, and service quality (Table 3).

**Table 3:** Proposed stakeholder responsibilities and recommended actions for supporting reproductive-health-related access.

Stakeholder	Primary role	Recommended actions
Ministry of Health and Population	Policy formulation, regulation, and service provision	Integrate disability-inclusive reproductive health services into national health programs; support the provision of accessible AI-assisted technologies; establish standards for privacy and accessibility.
Healthcare Facilities and Service Providers	Delivery of reproductive healthcare services	Improve physical accessibility, ensure privacy during consultations, strengthen disability-sensitive practices, and provide appropriate reproductive health counseling for women with disabilities.
Technology Developers and Manufacturers	Design, production, and maintenance of assistive technologies	Develop affordable and user-centered technologies; expand technical support and maintenance services; strengthen data protection and cybersecurity measures.
Disability-Support Organizations and Civil Society Groups	Advocacy, support, and community engagement	Facilitate access to assistive technologies; provide training and awareness programs; represent the needs and experiences of women with disabilities in policy discussions.
Educational and Research Institutions	Knowledge generation and capacity building	Conduct research on disability, AI, and reproductive rights; evaluate intervention outcomes; develop evidence-based recommendations for policy and practice.
Religious, Media, and Community Organizations	Social awareness and cultural change	Promote positive perceptions of disability; address stigma and discrimination; support inclusive messages concerning reproductive rights, marriage, family life, and social participation.

The proposed intervention strategy should be implemented through coordinated efforts among healthcare institutions, disability-support organizations, policymakers, educational institutions, and technology developers. Because the identified barriers involve technological, economic, institutional, and socio-cultural dimensions, no single stakeholder can effectively address all challenges independently. Therefore, for effective implementation, collaboration and multi-sectoral strategies need to be employed to ensure the integration of technology innovation with health care accessibility. The proposed intervention strategy was not implemented or evaluated in the present study; therefore, its feasibility, acceptability, cost, and effectiveness should be examined through future participatory research and pilot interventions.

## 7 Limitations

When evaluating the outcomes of this research, some limitations should be taken into account. First, as the research used purposive sampling and included only eight women with disabilities, it is impossible to generalize the outcomes for a wider population. Second, all participants were never married, and none had children. Consequently, many of their accounts reflected anticipated future reproductive health needs rather than lived experiences of marriage, pregnancy, childbirth, contraception, or reproductive healthcare service use. The findings therefore cannot demonstrate that assistive technologies resulted in the actual exercise of reproductive rights.

Third, this study concentrated solely on women with physical or visual disabilities and therefore omitted other types of disabilities. The small number of participants also limited systematic comparisons between the two disability groups. Fourth, mothers or sisters were present during the interviews to provide communication, accessibility, or emotional support. Although participants’ own responses were prioritized, family presence may have affected privacy and participants’ willingness to discuss sensitive sexual and reproductive health matters openly.

Fifth, the research took place in one location, and the findings may differ across geographical settings with different social, economic, cultural, infrastructural, and healthcare conditions. Sixth, the research relied on participants’ self-reported experiences and perceptions rather than objective measures of reproductive health status, reproductive health outcomes, healthcare utilization, or technological effectiveness.

Finally, participants used a heterogeneous range of AI-supported, digitally enabled, sensor-supported, and technologically enhanced assistive devices. The exact device models, software specifications, and degree of AI integration were not independently verified. Accordingly, the findings should not be interpreted as demonstrating the effects of artificial intelligence specifically or as assuming that every reported technology incorporated AI.

The findings are context-specific, although they may be transferable to similar settings involving women with physical or visual disabilities under conditions of economic and institutional disadvantage. Further research should include larger and more diverse samples, different disability and marital-status groups, multiple geographical settings, and objective assessment of technology characteristics, healthcare utilization, and reproductive health outcomes.

## 8 Conclusion

This study examined the potential contribution of AI-supported and digitally enabled assistive technologies to the promotion of reproductive rights among women with physical and visual disabilities in Minya Governorate, Egypt. The findings suggest that technologies such as smart wheelchairs, prosthetic limbs, screen readers, smart canes, and AI-enhanced glasses may contribute to creating enabling conditions for reproductive rights promotion by improving access to healthcare services and resources, facilitating access to reproductive health information and educational messages, and supporting anticipated informed reproductive health decision-making. However, these findings do not demonstrate that reproductive rights or improved reproductive health outcomes were directly achieved through technology use.

At the same time, participants identified several barriers, including high costs, limited maintenance services, privacy concerns, infrastructural challenges, and persistent social stigma. Because all participants were never-married women, the findings primarily concern anticipated reproductive health needs and anticipated future reproductive decision-making rather than direct experiences related to marriage, pregnancy, childbirth, contraception, or family-planning services. Consequently, the findings should be interpreted as reflecting perceived opportunities, barriers, and expectations regarding reproductive rights rather than lived experiences of reproductive healthcare utilization.

Findings from the study are consistent with feminist views on how having access to certain resources can enable individuals to make decisions regarding their reproductive freedom (Correa and Petchesky, 1994). In the case of a woman’s ability to use AI-supported and digitally enabled assistive technologies, such technologies serve as an example of enabling resources that provide women with greater access to information and mobility and may support anticipated reproductive autonomy.

Technological, institutional, and social factors are all required to promote reproductive rights for women with disabilities. To improve access to AI-supported and digitally enabled assistive technologies, therefore, there is a need for improved access to reproductive health services that are disability-inclusive; better training of healthcare providers to interact effectively with women with disabilities; better protection of their privacy; better access to healthcare environments and transportation; and, importantly, increased efforts to reduce the stigma that prevents women with disabilities from being socially included in both community and healthcare settings.

From a policy perspective, reproductive rights among women who have disabilities need to go beyond the availability of assistive devices for their benefit. Increasing the availability of reproductive care facilities to make them accessible, making assistive devices affordable, and improving the skills of healthcare workers to work with these women could enable more effective use of technology by them.

## Data Availability

The raw data supporting the conclusions of this article will be made available by the authors, without undue reservation.
