# Supplementary material for: AI and digital assistive technologies and reproductive rights among women with disabilities in Egypt: a qualitative SWOT study
Source: Front Sociol. 2026 Aug 31;11:1925235. doi: 10.3389/fsoc.2026.1925235 (PMC13572294; doi:10.3389/fsoc.2026.1925235)
Supplement: Supplementary file 2 [file Table_1.DOCX]

**Supplementary Material 1. Semi-Structured Interview Guide**

| **Domain** | **Main interview questions** | **Optional prompts/probes documented in the manuscript** |
| --- | --- | --- |
| **Assistive technology use** | What assistive technology or technologies do you currently use? How long have you used them? How do they support you in your daily life? | Type of technology; smart wheelchair; prosthetic limb; screen reader; smart cane; Eyedaptic glasses; VoiceOver; mobility; navigation; text-to-speech access; information access |
| **Mobility and navigation** | Does the technology support your ability to move or navigate more independently? | Independent movement; reduced reliance on family members or caregivers; confidence in movement; navigation |
| **Potential healthcare access** | Does the technology create opportunities for you to reach healthcare facilities or obtain healthcare-related information? | Reaching healthcare facilities; leaving the home; physical accessibility; potential rather than confirmed healthcare use |
| **Environmental accessibility** | What physical or environmental barriers continue to limit your mobility or access to healthcare facilities? | Unpaved roads; inaccessible or uneven sidewalks; inaccessible pathways; limited accessible transportation; lack of disability-friendly public facilities |
| **Sources of reproductive-health information** | Where do you obtain information about reproductive health? | Mothers or family-based knowledge; healthcare centers; written materials; digital resources; screen-reader-accessible information; other information sources |
| **Technology and information access** | Does your assistive technology help you access reproductive-health information more independently? | Screen readers; VoiceOver; written materials; audio access; digital information; repeating or reviewing information without another person reading it |
| **Reproductive-health information needs among participants with physical disabilities** | What reproductive-health information do you need or expect to need? | Physical and reproductive changes; available reproductive-health services; safe motherhood; marriage and spouse selection; premarital screening; sexually transmitted infections |
| **Reproductive-health information needs among participants with visual disabilities** | What reproductive-health information do you need or expect to need? | Menstrual hygiene; onset and management of menstruation; protection from sexual assault and harassment; reproductive and sexual-health risks of female genital mutilation; early detection of reproductive-system diseases and breast tumors |
| **Anticipated reproductive-health decision-making** | Does improved mobility or access to information help you feel more prepared for future reproductive-health decisions? | Access to different information sources; verification of information through healthcare centers or additional sources; anticipated rather than lived decision-making; confidence in future decisions |
| **Digital privacy** | Do you have concerns about the privacy of personal information stored, transmitted, or accessed through digital or assistive technologies? | Who may access personal information; information stored or transmitted through devices; uncertainty about how to protect personal data; need for legal and institutional protection |
| **Healthcare confidentiality** | Do healthcare settings provide adequate privacy and confidentiality when reproductive-health matters are discussed? | Presence of unnecessary individuals in service rooms; transparency concerning examination procedures; management of personal health information; possible effects on disclosure of sensitive information |
| **Affordability** | Are the acquisition, repair, and maintenance costs of the assistive technologies affordable? | Purchase cost; repair cost; maintenance cost; inability to afford technologies independently; dependence on household financial resources |
| **Maintenance and technical support** | What difficulties do you experience when the technology requires repair or maintenance? | Device malfunction; limited availability of replacement parts; limited availability of qualified technicians; maintenance services |
| **Family support and dependency** | How does family support affect your ability to obtain, use, or maintain assistive technology? | Family financial support; household resources; communication and accessibility support; continued dependence on family financing; reduced mobility or information dependence but continuing financial dependence |
| **Healthcare-provider practices** | Do healthcare providers have the knowledge, communication skills, and professional competencies needed to support women with disabilities? | Disability-related knowledge; communication skills; reproductive-health education; counseling; privacy; professional support |
| **Social stigma** | What social attitudes or beliefs affect women with disabilities in relation to reproductive health, marriage, and motherhood? | Beliefs that women with disabilities cannot marry or have children; stigma; discrimination; social exclusion; poor treatment when seeking information or services |
| **Strengths of assistive technologies** | What are the main strengths of the assistive technologies you use? | Independent mobility; reduced reliance on assistance; information access; navigation; self-confidence; independence; interaction with educational resources |
| **Weaknesses of assistive technologies** | What are the main weaknesses or limitations of the assistive technologies you use? | High cost; maintenance requirements; limited spare parts; limited qualified technicians; data-privacy concerns; limited financial support |
| **Opportunities** | What opportunities may increase the usefulness of assistive technologies for reproductive-health-related access? | Potential access to healthcare facilities; reproductive-health information; self-education; interaction with healthcare providers; anticipated informed decision-making |
| **Threats** | What external barriers restrict the effective use of assistive technologies? | Inaccessible infrastructure; limited transportation; insufficient provider competencies; limited healthcare privacy; cultural stigma and discrimination |
